# THE LIFESTYLE AND UNMET NEEDS AMONG UNMARRIED MIDDLE-AGED AND OLDER ADULTS IN TAIWAN

**DOI:** 10.1093/geroni/igae098.3279

**Published:** 2024-12-31

**Authors:** Ching-Ju Chiu

**Affiliations:** National Cheng Kung University, Tainan, Tainan, Taiwan (Republic of China)

## Abstract

This study used in-depth interviews and questionnaires to explore the lifestyle and psychological state of Generation X singles in urban areas of Taiwan, using a mixed methodology that combines qualitative and quantitative research methods. The interview focuses on the individual’s understanding and planning of single life, as well as the acceptance of emerging technologies, while the questionnaire includes personality traits and loneliness tests to supplement the qualitative information in a quantitative way. This mixed research strategy maximizes the understanding of the target group, focusing on the target group’s preparation for future changes in the life stage of legal old age (65 years old) and their attitudes towards emerging technologies, and also provides a scientific basis for formulating more effective policies. After conducting in-depth conversations with multiple participants and understanding their life choices, expectations and challenges, the results of this study revealed the participants’ diverse attitudes towards single life and their flexible preparations for the future, as well as their acceptance of new technologies in daily life. It also provides many valuable insights to financial planners and government policy makers.

